# Prognostic values of *E2F* mRNA expression in human gastric cancer

**DOI:** 10.1042/BSR20181264

**Published:** 2018-12-21

**Authors:** Theasha Manicum, Fubiao Ni, Yiming Ye, Xuhui Fan, Bi-Cheng Chen

**Affiliations:** 1 Wenzhou Medical University,The First Affiliated Hospital of Wenzhou Medical University, Wenzhou, China; 2 Department of Hepatobiliary Surgery, The First Affiliated Hospital of Wenzhou Medical University, The Key Laboratory of Diagnosis and Treatment of Severe Hepato-Pancreatic Diseases of Zhejiang Province, Wenzhou, China; 3 Renji College of Wenzhou Medical University, Wenzhou, China; 4 The First Affiliated Hospital of Wenzhou Medical University, The Key Laboratory of Diagnosis and Treatment of Severe Hepato-Pancreatic Diseases of Zhejiang Province, Wenzhou, China

**Keywords:** E2F, gastric cancer, KM plotter, mRNA(mrna s), Prognostic values

## Abstract

Gastric cancer (GC) is the second most frequent cause of cancer-related mortality in the world, with Eastern Asia having the highest incidence rates. E2F is a family of transcription factor proteins that has a variety of functions, which include control of cell cycle, cell differentiation, DNA damage response and cell death. E2F transcription factors are divided into two subfamilies: transcription activators (E2F transcription factors 1 (E2F1), 2 (E2F2) and 3a (E2F3a)) and repressors (E2F3b, E2F transcription factors 4 (E2F4), 5 (E2F5), 6 (E2F6), 7 (E2F7) and 8 (E2F8)). Studies have demonstrated that E2F had prognostic significance in a number of cancers. However, the entirety of the prognostic roles of *E2F* mRNA expression in GC has not yet been apparently determined. In the present study, the prognostic value of individual family members of *E2F* mRNA expression for overall survival (OS) was evaluated by using online Kaplan–Meier Plotter (KM Plotter) database. Our result demonstrated that high expressions of three family members of E2F (E2F1, E2F3, E2F4) mRNA were significantly associated with unfavourable OS in all GC patients. However, increased expressions of E2F2, E2F5, E2F6 and E2F7 were significantly associated with favourable OS, especially for higher clinical stages in GC patients. These results provided a better insight into the prognostic functions of *E2F* mRNA genes in GC. Although the results should be further verified in clinical trials, our findings may be a favourable prognostic predictor for the development of newer therapeutic drugs in the treatment of GC.

## Introduction

Gastric cancer (GC) is the second most frequent cause of cancer-related mortality in the world, with Eastern Asia having the highest incidence rates [1]. Gastric adenocarcinoma (GAC) is the most common type of GC and according to the Lauren Classification, it is classified into two histological types: intestinal and diffuse [2]. Investigations and attempts at identifying molecular target therapy to improve patients’ outcomes still continue, thus GC remains a challenge to cure [3]. There have been various chemotherapeutic agents, which have been found to improve survival, however the median survival still remains less than a year [4]. Therefore, the identification of prognostic markers in GC is fundamental in improving clinical outcomes for GC patients.

E2F is a family of transcription factor proteins that has a variety of functions and has earned its title as a master of regulators of cell proliferation. Its variety of functions include, control of cell cycle, cell differentiation and DNA damage response and cell death [5,6]. This transcription factor family also consists of DNA-binding domains (DBD), which binds to specific target promoters and regulates their expressions [7,8]. E2F transcription factors are divided into two subfamilies: transcription activators (E2F transcription factors 1 (E2F1), 2 (E2F2) and 3a (E2F3a)) and repressors (E2F transcription factors 3b (E2F3b), 4 (E2F4), 5 (E2F5), 6 (E2F6), 7 (E2F7) and 8 (E2F8)) [6]. The first six E2F transcription factors also bind to DNA as heterodimers with the related dimerisation proteins (DP), such as DP1 and DP2 [5,6]. Transcription factors E2F7 and E2F8 are unique, because they consist of two unique DNA-binding subdomains [9]. E2F repressors that function independently from retinoblastoma (Rb), may form a new class of E2Fs (E2F6 and E2F7), and this is due to the evolution of these repressors [10]. Quiescent cells can be driven into S-phase via activator transcription factors, E2F1, E2F2 and E2F3a, by activating target genes required for G_1_/S transition [11]. During G_0_ phase, repressors E2F4 and E2F5 that are bound to Rb-related pocket proteins and associated co-repressors, supress target gene transcription. E2F6 contains MAX gene associated (Mga) and MYC-associated factor X (Max) which are part of a multimeric protein complex that represses G_1_/S gene transcription, independent from Rb Family members [12,13].

In most human cancers E2F transcription factors are altered and deregulated, via different molecular mechanisms that inactivate the Rb family [14]. E2F family member activators could possess oncogenic behaviour in human carcinogenesis. Repressors from the E2F family could be associated with tumour suppressing functions in human carcinogenesis [6].

Research has revealed that E2F1’s overexpression in GC prompted an outspread of cell death through various mechanisms, therefore proving the role of E2F1 in tumour suppression in GC [15]. However, the entirety of the prognostic roles of *E2F* mRNA expression in GC has not been determined apparently. In the current study, we will investigate whether E2F genes are of prognostic significance in human GC patients. We will evaluate clinical data, which include clinical stages, Lauren classification, differentiation degree, human epidermal growth factor receptor-2 (HER2) status and gender and treatment strategies. In the present study, we comprehensively investigated the prognostic values of seven E2F family members using the Kaplan–Meier Plotter (KM Plotter).

## Methods

The prognostic values for individual E2Fs members’ mRNA expressions for overall survival (OS) were evaluated by using online KM Plotter (http://kmplot.com/analysis) database. This database was established using gene expression data and survival information from Gene Expression Omnibus (GEO) [16], including Genet Sel Evol (GSE)14210 [17], GSE22377 [18], GSE51105 [19], GSE15459 [20] and GSE29272 [21]. Currently, the database has been established with 54675 genes that have been identified in GC [16], breast cancer [22], ovarian cancer [23], lung cancer [24] and liver cancer [25]. The database consists of a collection of clinical data including Lauren classification, clinical stages, differentiation degree, gender, HER2 status and treatment of GC patients. In the present study, using the available data source, we gathered the clinical data. We entered seven individual members of the E2F family (E2F1, E2F2, E2F3, E2F4, E2F5, E2F6, E2F7), to obtain Kaplan–Meier survival plots. Hazard ratio (HR), 95% confidence interval (CI) and log rank *P* were determined and displayed on the webpage. A value of *P*<0.05 was considered statistically significant.

## Results

### Prognostic values of *E2F* mRNA expression in all GC patients

In the current study, seven out of the eight E2F members’ data were obtained using the Kaplan–Meier survival plots (http://www.kmplot.com).

**Figure 1:**
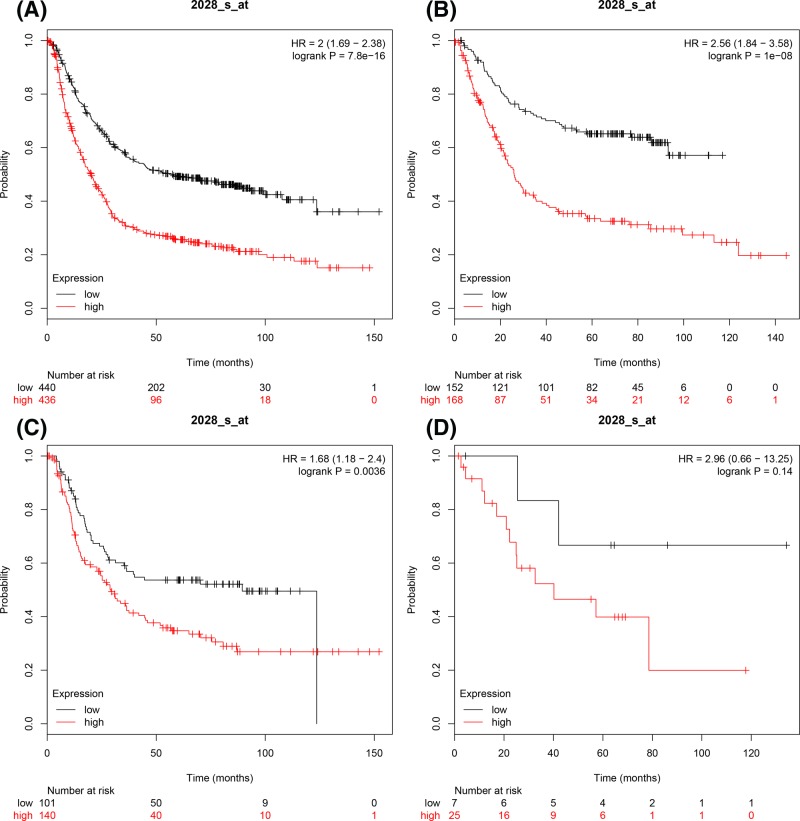
The prognostic values of E2F1 expression in GC The prognostic of E2F1 expression in www.kmplot.com ID. 2028_s_at. OS curves were plotted for (**A**) all the patients (*n*=876), (**B**) intestinal cancer patients, (**C**) diffuse cancer patients, (**D**) mixed cancer patients.

The prognostic values of *E2F1* mRNA expression were evaluated in the database. ID 2028_s_at. OS curves were plotted for GC patients. Increase in *E2F1* mRNA expression level revealed a significant association with poor OS, for all GC patients, (*n*=876), HR = 2 (1.69–-2.38), *P*=7.8 × 10^−16^ (Figure 1A). The Lauren classification subtype results revealed that increased *E2F1* mRNA expression was correlated with unfavourable OS for patients with intestinal GC, HR = 2.56 (1.84–3.58), *P*=1 × 10^−8^ (Figure 1B) and patients with diffuse GC, HR = 1.68 (1.18–2.4), *P*=0.0036 (Figure 1C). However, the expression level of *E2F1* mRNA in patients with mixed-type GC, did not show a significant correlation, HR = 2.96 (0.66–13.25), *P*=0.14 (Figure 1D).

**Figure 2:**
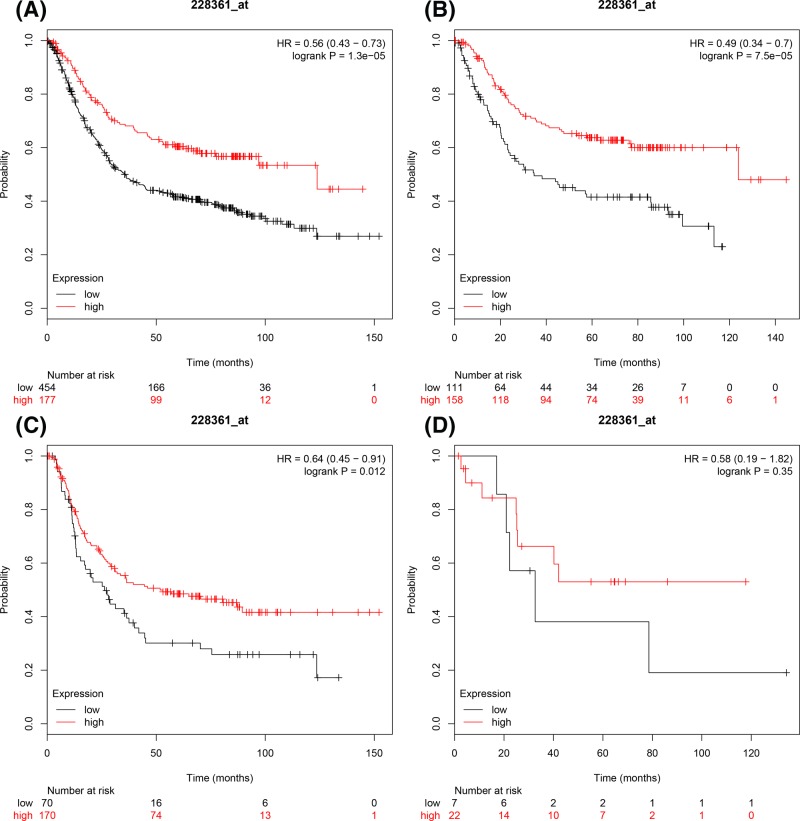
The prognostic values of E2F2 expression in GC The prognostic of E2F2 expression in www.kmplot.com ID.228361_at. OS curves were plotted for (**A**) all the patients (*n*=876), (**B**) intestinal cancer patients, (**C**) diffuse cancer patients, (**D**) mixed cancer patients.

The next set of prognostic values for *E2F2* mRNA expression were evaluated in the database. ID228361_s_at. *E2F2* mRNA expression levels were significantly associated with favourable OS for all GC patients, HR = 0.56 (0.43–0.73), *P*=1.3 × 10^−5^ (Figure 2A), intestinal GC patients, HR = 0.49 (0.34–0.7), *P*=7.5 × 10^−5^ (Figure 2B), and diffuse GC patients, HR = 0.64 (0.45–0.91), *P*=0.012 (Figure 2C). Whereas the expression level of *E2F2* mRNA in mixed-type GC patients did not show any association with OS, HR = 0.58 (0.19–1.82), *P*=0.35 (Figure 2D).

**Figure 3:**
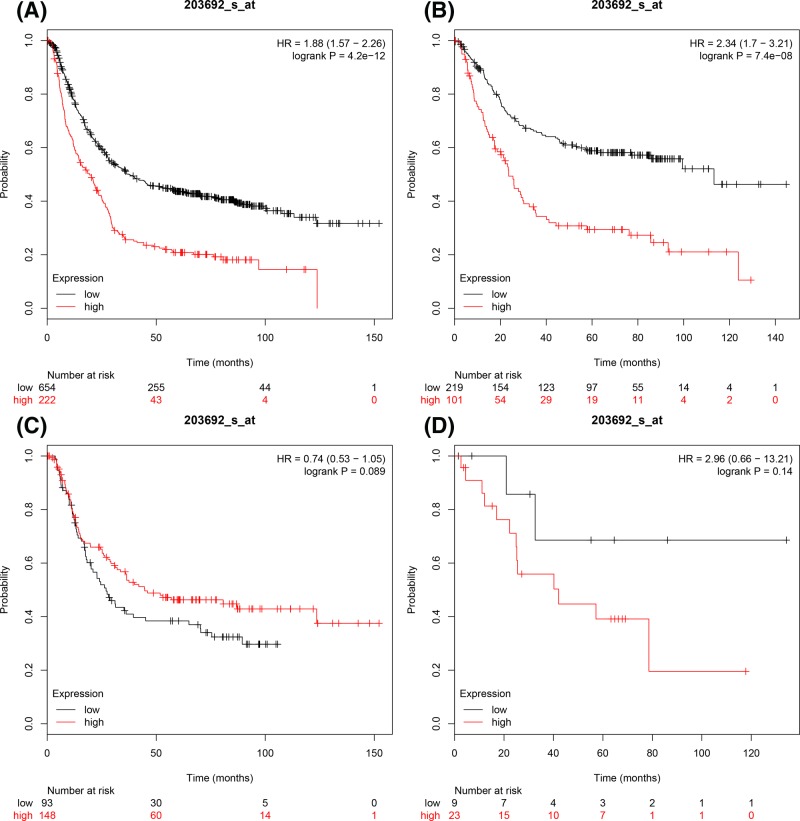
The prognostic values of E2F3 expression in GC The prognostic of E2F3 expression in www.kmplot.com ID. 203692_s_at. OS curves were plotted for (**A**) all the patients (*n*=876), (**B**) intestinal cancer patients, (**C**) diffuse cancer patients, (**D**) mixed cancer patients.

Figure 3 showed the prognostic values of E2F3 in the database. ID 203692_s_at. *E2F3* mRNA expression was significantly associated with poor OS for all GC patients and intestinal cancer patients, HR = 1.88 (1.57–2.26), *P*=4.2 × 10^−12^ (Figure 3A), HR = 2.34 (1.7–3.21), *P*=7.4 × 10^−8^ (Figure 3B) respectively. However, there was no significant association in OS of diffuse GC patients and mixed type GC patients, HR = 0.74 (0.53–1.05), *P*=0.089 (Figure 3C), HR = 2.96 (0.66–13.21), *P*=0.14 (Figure 3D) respectively.

**Figure 4:**
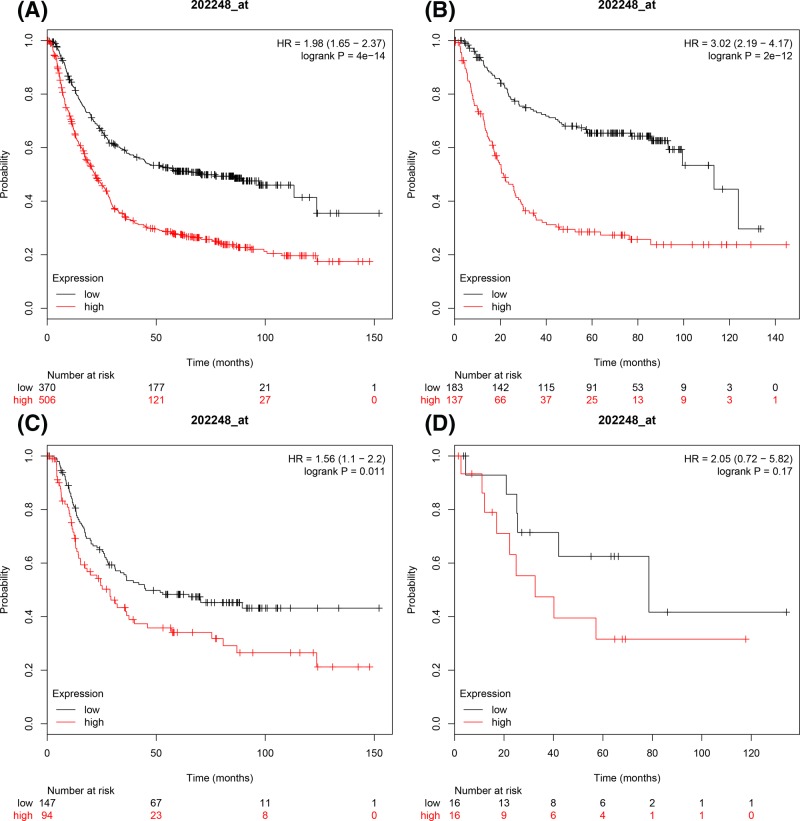
The prognostic values of E2F4 expression in GC The prognostic of E2F4 expression in www.kmplot.com ID. 202248_at. OS curves were plotted for (**A**) all the patients (*n*=876), (**B**) intestinal cancer patients, (**C**) diffuse cancer patients, (**D**) mixed cancer patients.

Figure 4 presented prognostic significance of *E2F4* mRNA expression in the database. ID 202248_s_at. High *E2F4* mRNA levels were significantly correlated with unfavourable OS in all GC patients, intestinal GC patients and diffuse GC patients, HR = 1.98 (1.65–2.37), *P*=4 × 10^−14^ (Figure 4A), HR = 3.02 (2.19–4.17), *P*=2 × 10^−12^ (Figure 4B), HR = 1.56 (1.1–2.2), *P*=0.011 (Figure 4C) respectively. But there was not any association with OS of mixed type GC, HR = 2.05 (0.72–5.82), *P*=0.17 (Figure 4 D).

**Figure 5:**
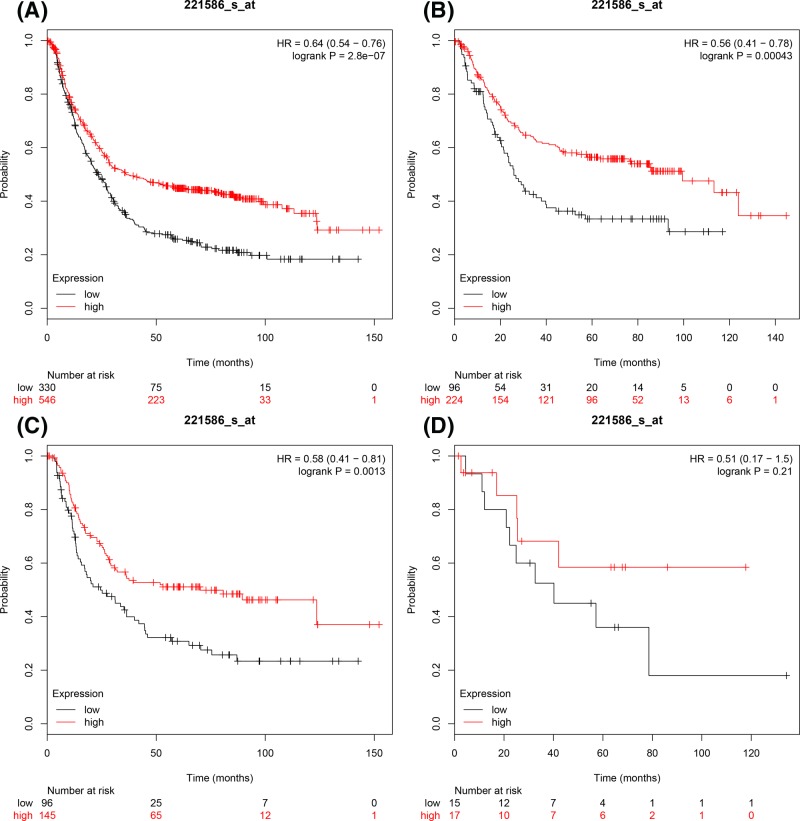
The prognostic values of E2F5 expression in GC The prognostic of E2F5 expression in www.kmplot.com ID. 221586_s_at. OS curves were plotted for (**A**) all the patients (*n*=876), (**B**) intestinal cancer patients, (**C**) diffuse cancer patients, (**D**) mixed cancer patients.

Figure 5 illustrated prognostic association of *E2F5* mRNA expression in the database. ID 221586_s_at. Increased expression of E2F5 was correlated with favourable OS in all GC patients, HR = 0.64 (0.54–0.076), *P*=2.8 × 10^−7^ (Figure 5A), intestinal cancer patients, HR = 0.56 (0.41–0.76), *P*=0.00043 (Figure 5B) and diffuse GC patients, HR = 0.58 (0.41–0.81), *P*=0.0013 (Figure 5C). However, increased *E2F5* mRNA in mixed-type mRNA was not correlated with OS, HR = 0.51 (0.17–1.5), *P*=0.21 (Figure 5D).

**Figure 6:**
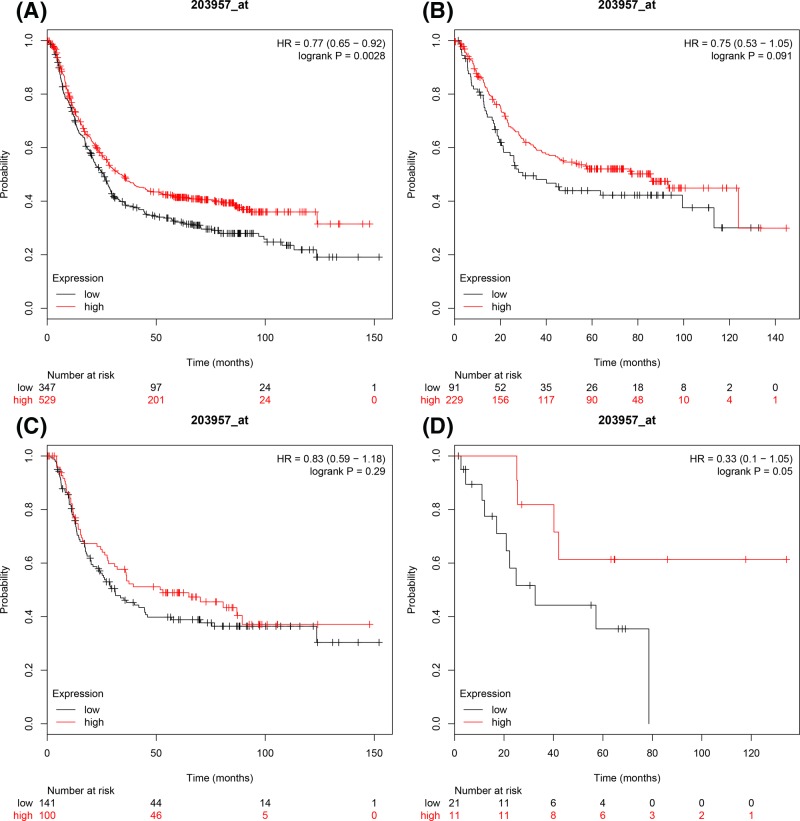
The prognostic values of E2F6 expression in GC The prognostic of E2F6 expression in www.kmplot.com ID. 203957_at. OS curves were plotted for (**A**) all the patients (*n*=876), (**B**) intestinal cancer patients, (**C**) diffuse cancer patients, (**D**) mixed cancer patients.

Prognostic values for *E2F6* mRNA expression were evaluated in the database. ID 203957_s_at. Overexpression of *E2F6* mRNA was found to be associated with favourable OS of all GC patients, HR = 0.77 (0.65–0.92), *P*=0.0028 (Figure 6A) and mixed-type GC, HR = 0.33 (0.1–1.05), *P*=0.05 (Figure 6D). Whereas, increased *E2F6* mRNA expression showed no correlation with OS in neither intestinal GC, HR = 0.75 (0.53–1.05), *P*=0.091 (Figure 6B) nor diffuse GC, HR = 0.83 (0.59–1.18), *P*=0.29 (Figure 6C).

**Figure 7:**
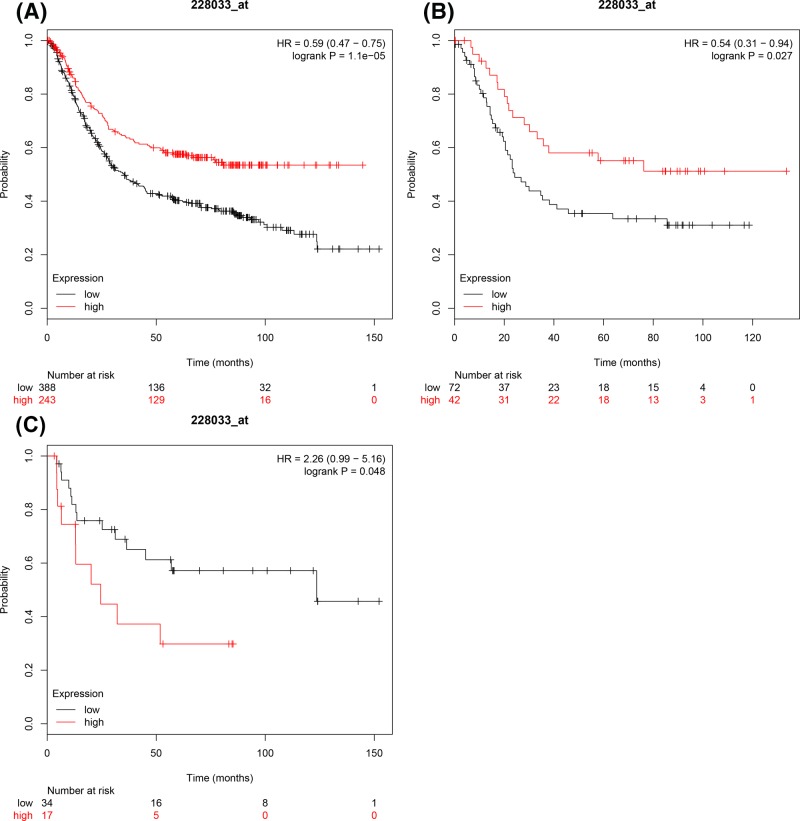
The prognostic values of E2F7 expression in GC The prognostic of E2F7 expression in www.kmplot.com ID.228033_s_at. OS curves were for (**A**) all the patients (*n*=876), (**B**) intestinal cancer patients, (**C**) diffuse cancer patients.

Figure 7 demonstrated prognostic significance of *E2F7* mRNA expression in the database. ID 228033_s_at. High expression of *E2F7* mRNA levels showed a significant correlation with favourable OS in all GC patients, HR = 0.59 (0.47–0.75), *P*=1.1 × 10^−5^ (Figure 7A) and intestinal GC patients, HR = 0.54 (0.31–0.94), *P*=0.027 (Figure 7B). However, overexpression of *E2F7* mRNA showed a signification association with unfavourable OS in diffuse GC patients, HR = 2.26 (0.99–5.16), *P*=0.048 (Figure 7C) and mixed-type GC patients’ sample size was too small to demonstrate any significant results.

In addition to our investigations of the prognostic values of *E2F* mRNA expression, we evaluated the association with other clinicopathological characteristics, including correlation of E2Fs with clinical stages, HER2 status, treatment strategies and gender status and differentiation degree of GC patients.

As illustrated in Table 1, we found that overexpressions of E2F2, E2F5 and E2F6 were correlated with favourable OS in stage III GC patients. Consecutive mRNA expressions were associated with better OS in E2F5 stages (II, III and IV) and E2F7 stage IV GC patients. However, increased expressions in E2F1 all stages (I, II, III, IV), E2F3 stage III and E2F4 stage (III, IV) were correlated with poor OS in GC patients.

**Table 1:** Correlation of E2Fs’ genes expression with OS in GC patients at different clinical stages

E2Fs	Clinical stage	Cases	HR (95% CI)	*P*-value
E2F1	I	67	5.18 (1.45−18.45)	0.0049*
	II	140	2.19 (1.14−4.21)	0.016*
	III	305	2.29 (1.57−3.33)	9.9e−06*
	IV	148	1.55 (1.05−2.3)	0.027*
E2F2	I	62	0.43 (0.14−1.34)	0.13
	II	135	0.67 (0.36−1.24)	0.2
	III	297	0.53 (0.36−0.8)	0.0018*
	IV	135	0.67 (0.36−1.24)	0.2
E2F3	I	67	2.41 (0.89−6.49)	0.074
	II	140	1.77 (0.95−3.32)	0.07
	III	148	1.81 (1.36−2.41)	4e−05*
	IV	148	1.24 (0.83−1.84)	0.29
E2F4	I	67	2.48 (0.92−6.66)	0.064
	II	140	1.73 (0.93−3.21)	0.078
	III	305	2.23 (1.6−3.11)	1.1e−06*
	IV	148	1.64 (1.11−2.43)	0.012*
E2F5	I	67	0.57 (0.2−1.64)	0.29
	II	140	0.52 (0.28−0.97)	0.036*
	III	305	0.63 (0.47−0.84)	0.0016*
	IV	148	0.55 (0.35−0.84)	0.0051*
E2F6	I	67	0.44 (0.16−1.18)	0.095
	II	140	1.77 (0.96−3.24)	0.063
	III	305	0.74 (0.56−0.98)	0.037*
	IV	148	0.73 (0.49−1.07)	0.1
E2F7	I	62	0.3 (0.08−1.11)	0.056
	II	135	0.55 (0.29−1.04)	0.063
	III	197	0.63 (0.41−0.96)	0.032*
	IV	140	0.57 (0.37−0.86)	0.007*

* *P*<0.05.

In Table 2 we further investigated the association between E2Fs expression and HER2 status in GC patients. E2F2 and E2F5 showed correlations with better OS when HER2 expression was both positive and negative in GC patients. A negative expression of HER2 in E2F6 and E2F7 was correlated with favourable OS in GC patients. However, both positive and negative HER2 expressions were associated with unfavourable OS in *E2F1, E2F3* and *E2F4* mRNA expressions in GC patients. On the other hand, E2F6 and E2F7 had no correlation with positive HER2 status in GC patients.

**Table 2:** Correlation of E2Fs’ genes expression with OS in GC patients with HER2 expression status

E2Fs	HER2 status	Cases	Low	High	HR (95% CI)	*P-value*
E2F1	Negative	532	292	240	1.98 (1.58−2.48)	1.7e−09*
	Positive	344	131	213	1.93 (1.46−2.55)	2.7e−06*
E2F2	Negative	429	222	207	0.6 (0.46−0.79)	0.00018*
	Positive	202	115	87	0.6 (0.4−0.88)	0.0083*
E2F3	Negative	532	361	171	1.64 (1.3−2.07)	2.1e−05*
	Positive	344	185	159	1.76 (1.36−2.29)	1.6e−05*
E2F4	Negative	532	292	240	1.84 (1.47−2.3)	8.1e−08*
	Positive	344	120	224	2.01 (1.49−2.7)	2.5e−06*
E2F5	Negative	532	192	340	0.58 (0.46−0.72)	1.5e−06*
	Positive	344	126	218	0.71 (0.55−0.93)	0.011*
E2F6	Negative	532	201	331	0.69 (0.55−0.86)	0.0012*
	Positive	344	156	188	0.89 (0.68−1.15)	0.36
E2F7	Negative	429	154	275	0.54 (0.41−0.7)	3.7e−06*
	Positive	202	142	60	0.65 (0.43−1.01)	0.051

* *P*<0.05.

The results in Table 3 showed the correlation of *E2F* mRNA expression with OS using different treatment strategies in GC patients. The results revealed that expressions of E2F2 and E2F7 showed better OS for GC patients treated with surgery alone. However, increased mRNA expressions of E2F1, E2F3 and E2F4 were significantly correlated with poor OS for GC patients treated with surgery alone. Consecutive expressions of all E2Fs except E2F2 showed unfavourable OS in GC patients treated with 5-Fluorouracil (5FU) adjuvant treatment. Subsequent expressions of E2F2, E2F4 and E2F5 were significantly associated with favourable OS, when GC patients were treated with other adjuvant treatments.

**Table 3:** Correlation of E2Fs’ genes expression with OS in GC patients with different treatment strategies

E2Fs	Treatment	Cases	HR (95% CI)	*P*-value
E2F1	Surgery alone	380	1.45 (1.06−1.97)	0.019*
	5FU-based adjuvant	153	2.06 (1.36−3.13)	0.00054*
	Other adjuvant	76	0.56 (0.23−1.37)	0.2
E2F2	Surgery alone	380	0.59 (0.42−0.83)	0.0022*
	5FU-based adjuvant	34	2.47 (0.94−6.44)	0.058
	Other adjuvant	76	0.4 (0.16−0.99)	0.041*
E2F3	Surgery alone	380	1.43 (1.05−1.95)	0.023*
	5FU-based adjuvant	153	1.8 (1.24−2.62)	0.0018*
	Other adjuvant	76	0.44 (0.18−1.08)	0.064
E2F4	Surgery alone	380	1.41 (1.02−1.93)	0.034*
	5FU-based adjuvant	153	1.81 (1.22−2.68)	0.0026*
	Other adjuvant	76	0.26 (0.1−0.73)	0.0056*
E2F5	Surgery alone	380	0.76 (0.56−1.04)	0.085
	5FU-based adjuvant	153	1.85 (1.27−2.7)	0.0013*
	Other adjuvant	76	0.26 (0.1−0.67)	0.0028*
E2F6	Surgery alone	380	0.85 (0.62−1.17)	0.31
	5FU-based adjuvant	153	1.78 (1.24−2.55)	0.0015*
	Other adjuvant	76	3.33 (0.77−14.37)	0.087
E2F7	Surgery alone	380	0.64 (0.47−0.86)	0.0033*
	5FU-based adjuvant	34	3.24 (1.26−8.28)	0.01*
	Other adjuvant	76	0.51 (0.2−1.27)	0.14

* *P*<0.05.

Table 4 showed prognostic significance between the *E2F* mRNA expression and gender in GC patients. Increased expressions of E2F2, E2F5 and E2F7 were correlated with favourable OS in both male and female GC patients. However, overexpressions of E2F1 and E2F4 were associated with poor OS for both male and female GC patients. Subsequent expression of E2F6 was significantly correlated with favourable OS for male patients, whereas E2F3 expression was correlated with unfavourable OS for female GC patients.

**Table 4:** Correlation of E2Fs’ genes expression with OS in GC patients with gender expression status

E2Fs	Gender	Cases	HR (95% CI)	*P*-value
E2F1	Male	545	2.33 (1.84−2.95)	5.1e−13*
	Female	876	2 (1.69−2.38)	7.8e−16*
E2F2	Male	349	0.53 (0.39−0.72)	2.6e−05*
	Female	187	0.53 (0.31−0.91)	0.02*
E2F3	Male	545	2.08 (1.68−2.58)	9e−12*
	Female	236	1.54 (1.05−2.25)	0.026
E2F4	Male	545	2.24 (1.79−2.81)	7.2e−13*
	Female	236	2.17 (1.53−3.09)	8.6e−06*
E2F5	Male	545	0.65 (0.52−0.81)	9e−05*
	Female	236	0.59 (0.42−0.84)	0.003*
E2F6	Male	545	0.89 (0.71−1.11)	0.28
	Female	236	0.56 (0.39−0.79)	0.00097*
E2F7	Male	349	0.6 (0.45−0.82)	0.0011*
	Female	187	0.48 (0.3−0.75)	0.0011*

* *P*<0.05.

Lastly, Table 5 demonstrated correlations of E2Fs expression with OS according to their differentiation degree in GC patients. We found that GC patients with high expressions of E2F2 and E2F3 with moderate differentiation had better OS. Whereas, E2F3 and E2F7 expressions correlated with worse OS for poor differentiation and E2F4 expression was also correlated with worse OS for moderate differentiation in GC patients. All other gene expressions showed no significance in OS with differentiation degree in GC patients.

**Table 5:** Correlation of E2Fs’ genes expression with OS in GC patients with differentiation degree

E2Fs	Differentiation	Cases	HR (95% CI)	*P*-value
E2F1	Poor	165	1.43 (0.91−2.26)	0.12
	Moderate	67	0.63 (0.29−1.39)	0.25
	Good	32	1.87 (0.77−4.54)	0.16
E2F2	Poor	121	0.65 (0.34−1.24)	0.19
	Moderate	67	0.37 (0.19−0.74)	0.0037*
	Good	_	_	_
E2F3	Poor	165	1.53 (1.01−2.3)	0.042*
	Moderate	67	0.49 (0.26−0.95)	0.031*
	Good	32	2.47 (0.72−8.47)	0.14
E2F4	Poor	165	1.3 (0.86−1.95)	0.21
	Moderate	67	3.98 (1.51−10.48)	0.0028*
	Good	32	3.06 (0.9−10.42)	0.06
E2F5	Poor	165	1.45 (0.97−2.15)	0.066
	Moderate	67	0.75 (0.37−1.55)	0.44
	Good	32	0.59 (0.25−1.39)	0.22
E2F6	Poor	165	1.43 (0.95−2.15)	0.084
	Moderate	67	2.05 (1.05−4.01)	0.032
	Good	32	0.71 (0.3−1.7)	0.44
E2F7	Poor	121	2.12 (1.27−3.52)	0.0032*
	Moderate	67	0.68 (0.35−1.35)	0.27
	Good	_	_	_

* *P*<0.05.

## Discussion

In the current study, we investigated the prognostic significance of *E2F* mRNA expression in human GC and we gathered all our data by using the KM Plotter. Our results revealed that, E2F2, E2F5, E2F6 and E2F7 family members were significantly associated with favourable survival in all GC patients. However, overexpressions of E2F1, E2F3 and E2F4 revealed unfavourable OS in all GC patients.

Among all the E2F family members E2F1 has been the most researched and investigated member in human cancers [26]. Numerous studies have reported that E2F1 expression was significant for poor prognosis in malignancies such as oesophageal carcinoma [27], hepatic cellular carcinoma (HCC) [28], pancreatic cancer [29], non-small cell lung cancer [30] and breast cancer [31]. However, previous studies indicated that E2F1 played a tumour suppressing role in GC, and this was due to apoptosis being induced by adenovirus-mediated overexpression of E2F1. The cell death is probably due to a combination of immunologic signalling pathways and cyclin-dependent kinase (CDK) inhibitors [15,32–33]. In another study done by Xu et al. [34], it was discovered that overexpression of E2F1 played a role in cell growth and tumorigenicity. These patients’ higher expression of E2F1 was shown to have a poor survival, due to increased tumour sizes and higher tumour stages. This was similar to discoveries made in our study, in which overexpression of *E2F*1 mRNA was associated with unfavourable OS in all clinical stages and both male and female GC patients.

E2F2 was critical to many cell processes, including the cell cycle, proliferation, differentiation and cancer development [35–37]. It has recently been reported that in colorectal adenocarcinoma [38], E2F2 expression at the tissue level was low. Li et al. [46] indicated that E2F2 promoter polymorphisms, which affected the expression of E2F2, were significantly associated with increased risk squamous cell carcinoma of the oropharynx and many other cancers. E2F2 has not been investigated in GC. In our current analysis, we have found that high E2F2 expression was significantly correlated with favourable survival, especially with higher clinical stages (stages III) in GC patients.

According to previous studies, high expression of E2F3 has been known to show poor prognosis in many human cancers such as human bladder and prostate cancer. E2F3 has been known to be significant with tumour stages, grades and cell proliferation in bladder cancer and significant with tumour aggressive in prostate cancer [39]. Zeng et al. [40] found that E2F3 was also correlated with an unfavourable biomarker in HCC patients and high expression indicated a poor prognosis. In the present study, we have discovered that E2F3 had also been correlated with the evidence of unfavourable prognosis, especially with higher clinical stages (stages III) in GC patients.

In earlier investigations it has been demonstrated that E2F4 mutations had been associated with GACs, ulcerative colitis-associated neoplasms, colorectal carcinomas, endometrial cancers and prostatic carcinomas and that E2F4 expression did not assist in apoptosis [41]. Patients with breast cancer exhibiting increased expression of E2F4 target genes displayed a more severe cancer and shorter survival [44]. The changes in the levels of E2F4 in prostate cancer were thought to promote increased sensitivity, via inactivation of E2F4 by siRNA, and prevent ionising radiation-induced apoptosis [42,43]. Farman et al. [45] displayed that E2F4 methylation status had a noteworthy influence on its expression, and that there might be some prognostic values in breast carcinogenesis. In our study’s results, we have discovered that high E2F4 expression was significantly correlated with poor OS in all the patients with GC.

Recent studies have shown that E2F5 expression was associated with several tumours, such as glioblastoma [47], prostate cancer [48] and Rb [49]. Fang et al. [47] reported that due to the down-regulation of E2F5 expression, miR-129-3p was inhibited in glioblastoma. Zhang et al. [49] indicated that miR-613 functioned as a tumour suppressor in Rb through down-regulation of E2F5. In human lung carcinoma E2F5 expression was reportedly increased and was significantly associated with worse OS [50]. Nevertheless, it has not been reported in GC. In the present study, we demonstrated that higher expression of E2F5 in GC patients had better prognosis, and this expression was associated with clinical stages (stages II, III, IV) in patients.

Numerous studies have reported that E2F6 expression was significant for prognosis in malignancies such as pancreatic cancer [51], breast cancer [52] and nasopharyngeal carcinoma [53]. What is more, E2F6 functions as a tumour suppressor in nasopharyngeal carcinoma. Restoring E2F6 expression in nasopharyngeal carcinoma impairs proliferation [53]. However, the prognostic role of E2F6 in GC has yet to be investigated. We have also discovered similar findings in that high E2F6 expression was significantly correlated with favourable OS in all the patients with GC, and this expression was apparently correlated with clinical stages (stage III) in patients.

Various previous studies have shown that E2F7 was associated with several tumours, such as gallbladder cancer [54], gliomas [55] and squamous cell carcinoma [56]. E2F7 was also associated with breast cancer, and increased expression of E2F7 was significantly correlated with worse prognosis in patients being treated with tamoxifen [57]. In GC, the prognostic role of E2F7 is yet to be investigated. Therefore, in the present study, we demonstrated that high E2F7 expression was significantly correlated with better OS in all the patients with GC. This expression was markedly correlated with higher clinical stages (stages III, IV) in GC patients.

HER2 belongs to the epidermal growth factor receptor (EGFR) family. HER2 expression is associated with poor prognosis in GC, which is also a predictive factor of poor response to hormonal therapy and chemotherapy [58]. HER2 positive tumours resulted from E2Fs activity and involvement, therefore it could be used as a tool to predict relapse-free survival [59]. According to our tests, negative HER2 expression of E2F6 and E2F7 was associated with favourable prognosis in GC patients. Besides, the theory needs to be further tested. 5FU has been used for a number of clinical applications including cancer therapy and antiproliferative treatment, and it is most commonly used to treat gastrointestinal cancers such as colorectal cancer, GAC and pancreatic cancer [60]. According to a study by Yu et al. [61], GC cells have some resistance to 5FU in the presence of certain molecular mechanisms and high expressions of certain target genes. In our study, the data clearly indicated that higher E2F expression was significantly correlated with poor prognosis in patients treated with 5FU adjuvant treatment. Thus, we recommend that 5FU-based adjuvant therapy should be avoided in GC patients, who have higher E2F expression.

## Conclusion

In the current study the prognostic values of mRNA expression of E2F family members in human GC was analysed using the KM Plotter. Our result demonstrated that three family members of E2F (E2F1, E2F3, E2F4) mRNA expressions were significantly associated with unfavourable OS in GC patients. However, increased expressions of E2F2, E2F5, EF6 and E2F7 were significantly associated with favourable OS, especially for higher clinical stages in GC patients. In addition to our investigation, we have observed that high expressions of E2F2, E2F5, E2F6 and E2F7 suggested favourable prognosis under HER2 negative status and E2F2 and E2F5 also suggested favourable prognosis under HER2 positive status. In terms of treatment strategies in GC patients, our results showed that treatment with 5FU-based adjuvant was significant with unfavourable prognosis in E2F family members. According to our results, the prognostic values of *E2F* mRNA expression were significantly favourable, except for E2F1, E2F3 and E2F4 in GC patients. These results provided a better insight into the prognostic functions of *E2F* mRNA genes in GC. Although the results should be further verified in clinical trials, our findings may be a favourable prognostic predictor in the development of newer therapeutic drugs in the treatment of GC.

## Abbreviations

5FU: 5-Fluorouracil
CI: confidence interval
DP: dimerisation protein
E2F1: E2F transcription factor 1
E2F2: E2F transcription factor 2
E2F3: E2F transcription factor 3
E2F4: E2F transcription factor 4
E2F5: E2F transcription factor 5
E2F6: E2F transcription factor 6
E2F7: E2F transcription factor 7
GAC: gastric adenocarcinoma
GC: gastric cancer
GSE: Genet Sel Evol
HCC: hepatic cellular carcinoma
HER2: human epidermal growth factor receptor-2
HR: hazard ratio
KM Plotter: Kaplan–Meier Plotter
OS: overall survival
Rb: retinoblastoma
